# Effects of antioxidant-rich *Lactiplantibacillus* *plantarum* inoculated alfalfa silage on rumen fermentation, antioxidant and immunity status, and mammary gland gene expression in dairy goats

**DOI:** 10.1186/s40104-023-00977-3

**Published:** 2024-01-22

**Authors:** Yixin Zhang, Samaila Usman, Qiang Li, Fuhou Li, Xia Zhang, Luiz Gustavo Nussio, Xusheng Guo

**Affiliations:** 1 State Key Laboratory of Grassland Agro-ecosystems, School of Life Sciences, Lanzhou University, Lanzhou, 730000 PR China; 2 Probiotics and Life Health Institute, Lanzhou University, Lanzhou, 730000 PR China; 3 Probiotics and Biological Feed Research Centre, Lanzhou University, Lanzhou, 730000 PR China; 4 Department of Animal Science, Luiz de Queiroz College of Agriculture, University of São Paulo, Piracicaba, 13418-900 Brazil

**Keywords:** Alfalfa silage, Antioxidant activity, Gene expression, Goats, Immunity, Lactation

## Abstract

**Background:**

Milk synthesis in lactating animals demands high energy metabolism, which results in an increased production of reactive oxygen metabolites (ROM) causing an imbalance between oxidants and antioxidants thereby inducing oxidative stress (OS) on the animals. To mitigate OS and postpartum disorders in dairy goats and gain insight into the impact of dietary choices on redox status during lactation, a feeding trial was conducted using alfalfa silage inoculated with a high-antioxidant strain of *Lactiplantibacillus* *plantarum*.

**Methods:**

Twenty-four Guanzhong dairy goats (38.1 ± 1.20 kg) were randomly assigned to two dietary treatments: one containing silage inoculated with *L. plantarum* MTD/1 (RSMTD-1), and the other containing silage inoculated with high antioxidant activity *L. plantarum* 24-7 (ES24-7).

**Results:**

ES24-7-inoculated silage exhibited better fermentation quality and antioxidant activity compared to RSMTD-1. The ES24-7 diet elevated the total antioxidant capacity (T-AOC), superoxide dismutase (SOD), glutathione peroxidase (GSH-Px), and catalase (CAT) activities in milk, serum, and feces of lactating goats (with the exception of T-AOC in milk). Additionally, the diet containing ES24-7 inoculated silage enhanced casein yield, milk free fatty acid (FFA) content, and vitamin A level in the goats’ milk. Furthermore, an increase of immunoglobulin (Ig)A, IgG, IgM, interleukin (IL)-4, and IL-10 concentrations were observed, coupled with a reduction in IL-1β, IL-2, IL-6, interferon (IFN)-γ, and tumor necrosis factor (TNF)-α concentrations in the serum of lactating goats fed ES24-7. Higher concentrations of total volatile fatty acid (VFA), acetate, and propionate were observed in the rumen fluid of dairy goats fed ES24-7 inoculated silage. Moreover, the diet containing ES24-7 inoculated silage significantly upregulated the expression of nuclear factor erythroid 2 like 2 (*NFE2L2*), beta-carotene oxygenase 1 (*BCO1*), *SOD1*, *SOD2*, *SOD3*, *GPX2*, *CAT*, glutathione-disulfide reductase (*GSR*), and heme oxygenase 1 (*HMOX1*) genes in the mammary gland, while decreased the levels of NADPH oxidase 4 (*NOX4*), *TNF*, and interferon gamma (*IFNG*).

**Conclusions:**

These findings indicated that feeding *L. plantarum* 24-7 inoculated alfalfa silage not only improved rumen fermentation and milk quality in lactating dairy goats but also boosted their immunity and antioxidant status by modulating the expression of several genes related to antioxidant and inflammation in the mammary gland.

**Supplementary Information:**

The online version contains supplementary material available at 10.1186/s40104-023-00977-3.

## Introduction

Reactive oxygen metabolites (ROM) are natural byproducts of cellular processes, and their excessive accumulation lead to oxidative stress (OS) by disrupting the balance between oxidants and antioxidants [1, 2]. Ruminants can encounter ROM due to external factors like high temperature, ultraviolet radiation, and fungal toxins from silage [3, 4], while internally, lactation triggers the production of reactive oxygen species (ROS) like superoxide anion (${{\text{O}}}_{2}^{-})$, hydroxyl radical (·OH), and H_2_O_2_, causing imbalances between oxidant and antioxidant levels [4, 5]. The need for oxygen (as a key substrate for several intracellular biochemical reactions and ATP production) and energy during milk synthesis escalates ROM production, which compromises the immune system of lactating animals [2, 6], making them vulnerable to postpartum diseases like mastitis [7, 8]. This not only affects the animal’s health but also the quality of their products (milk and meat) consumed by humans. Additionally, the health and immunity of newborn animals rely on nutrients and immunoglobulins passed to them from the dam through colostrum [9]. Therefore, enriching the diet of lactating ruminants with antioxidants can help maintain redox balance, mitigate OS, and benefit both the animals and consumers of their products.

Recent developments in silage additives and livestock safety have revealed various antioxidant supplements capable of enhancing antioxidant activity and health-related attributes of silage, livestock, and their products. A previous study reported that dairy goats fed anthocyanin-rich purple corn stover silage exhibited improved free radicals scavenging capacity and superoxide dismutase (SOD) activity, and up-regulated antioxidant-related genes [10]. Similarly, meat-producing goats fed *Garcinia mangostana* L. peel powder diet exhibited improved growth performance and plasma glutathione peroxidase (GSH-Px) activity [11]. Furthermore, supplementing dams with *Moringa oleifera* leaf powder rich in bioactive compounds led to an improvement in milk yield, colostrum antioxidant status, and the growth rate of newborn goats [6]. These dietary antioxidant supplements can enhance animals’ antioxidant levels and immunity, and improve the quality of their products for consumers.

Silage as a form of lactic acid bacteria (LAB) fermented roughage [12, 13], is a critical feed resource for herbivorous livestock that maintains high yield and ensures safe and high-quality livestock products. Advances in probiotics research have shown that some LAB possess additional functions other than being responsible for silage fermentation. Maragkoudakis et al. [14] reported that *L. plantarum* (PCA 236) treatment modulated gut microbiota and polyunsaturated fatty acid content in goats’ milk without affecting animals’ antioxidant capacity and immune performance. More recently, Li et al. [15] found that silage inoculated with feruloyl esterase-producing LAB improved forage utilization efficiency and antioxidant capacity of dairy goats, as well as fortified the immunity status through increased immunoglobulins in the blood serum. Our previous studies screened LAB strains that secrete antioxidant enzymes resulting in improved fermentation quality and antioxidant enzyme activities, as well as reduced losses of α-tocopherol and β-carotene in alfalfa silage [16, 17]. However, it remains unknown whether feeding goats silages inoculated with these LAB strains could result in the accumulation of antioxidant enzymes and other molecules in their bloodstream and subsequently transmit them to the mammary glands, thereby improving the antioxidant capacity of goat milk. We hypothesized that feeding alfalfa silage enriched with antioxidant compounds could result in the accumulation of antioxidants in the goats’ physiological systems. To achieve this, we conducted a study evaluating the fermentation quality and antioxidant profile of alfalfa silage inoculated with *L. plantarum* 24-7 characterized by a high-antioxidant activity, as well as its effects on comparative rumen fermentation, antioxidant capacity in the blood serum and milk, blood serum immunity traits, and the expression of mammary gland genes associated with inflammatory and antioxidant activities in dairy goats.

## Materials and methods

### Ethical statement

All animal procedures in this experiment adhered to ethical guidelines and relevant regulations. The study received approval from the Animal Ethics Committee of the School of Life Sciences (No. 545/2001) at Lanzhou University (Gansu, China).

### Fresh forage and silages

Alfalfa (*Medicago sativa* L.) was mowed at approximately 10 cm above ground by a mechanical harvester in the early flowering period from an alfalfa field on June 21, 2021. The fresh forage was wilted to a dry matter (DM) content of approximately 400 g/kg and chopped to length of 2–4 cm using a forage harvester (9Z-3.0, Zhengzhou Darui Machinery Equipment Co., Ltd., Zhengzhou, China). The nutrient and chemical composition of the fresh forage were determined (Additional file 1: Table S1). The wilted forage was divided into two piles and treated with either the reference strain *L. plantarum* MTD/1 (RSMTD-1) or the experimental strain *L. plantarum* 24-7 (ES24-7) at an application rate of 1 × 10^5^ colony-forming units (CFU)/g fresh weight (FW) using a knapsack sprayer (20-L capacity), diluted in non-chloride water source under a temperature of below 30 °C. The sources and screening methods of the *L. plantarum* MTD/1 and *L. plantarum* 24-7 have been described previously [17]. The antioxidant activities of these two strains are presented in Additional file 1: Table S2. After applying the inoculants, the forages were wrapped in 4–6 layers of 25 µm thick moisture-proof wrapping film using a baling equipment (PT-DB, Jining Pangtai Machinery Co., Ltd., Jining, China). The wrapped bales (65 cm tall × 55 cm diameter × 60 kg weight) were stored under a dry shed until the commencement of the feeding trial, 30 d after ensiling.

### Fermentation, chemical analysis, and antioxidant capacity of silages

The silage samples from each treatment (in sextuplicate) were divided into 3 lots. Silage juice from the first lot was filtrated and used for the immediate determination of pH and organic acids as described previously [17]. The remaining filtrate was centrifuged (12,000 × *g*, 15 min, 4 °C) and frozen at −20 °C for the determination of ammonia nitrogen (NH_3_-N) [18], water-soluble carbohydrate (WSC) [19] and non-protein nitrogen (NPN) [20]. The thawed supernatant was also used for determining total antioxidant capacity (T-AOC, A015-3-1), and the enzymatic activities of SOD (A001-3-2), GSH-Px (A005-1-2), and catalase (CAT, A007-1-1) using commercial assay kits (Nanjing Jiancheng Bioengineering Institute, Nanjing, China) with strict adherence to the manufacturer’s instructions. Another lot of silage was dried in a thermostatic oven dryer for 72 h at 65 °C to estimate DM content. The oven-dried silage was ground to pass through a 1-mm sieve. The crude protein (CP; total nitrogen × 6.25) was determined according to AOAC [21]. Heat-stable α-amylase and sodium sulfite were used in determining the neutral detergent fiber (aNDF), followed by acid detergent fiber (ADF) determination according to the methods of Robertson and Van Soest [22] using Ankom fiber analyzer (A2000I, Ankom Technology, Fairport, NY, USA). A third lot of the silage was lyophilized using a Freeze Dryer (Labconco, Kansas City, MO, USA), and the ground samples were used to detect α-tocopherol and β-carotene. The saponification and extraction method for α-tocopherol and β-carotene was the same as described previously [17]. Concentrations of α-tocopherol and β-carotene were determined using Agilent high-performance liquid chromatography (HPLC, 1200, Agilent Technologies, Inc., Santa Clara, CA, USA) equipped with an ultraviolet detector with a Synergi Hydro-RP (250 mm × 4.60 mm, 4 μm, 80A, Phenomenex, Torrance, CA, USA) column (eluent: 1 mL/min, acetonitrile-dichloromethane-methyl alcohol 7:2:1 (v/v/v); temperature: 25 °C). Detection was conducted at 285 nm for α-tocopherol and 450 nm for β-carotene.

### Experimental animals, diets, and management

This experiment was conducted at Dingxi Longxing Agriculture and Animal Husbandry Co., Ltd. (35.43°N, 104.52°E, 2023 m above sea level), where the average temperature during the study period (July to September 2021) was 17.8 °C. Twenty-four mid-lactation Guanzhong dairy goats, 2–3 years old with a mean body weight (BW) of 38.1 ± 1.20 kg at 120–132 days in milk (DIM) and milk yield of 1.40 ± 0.17 kg/d were used. They were randomly assigned to 2 experimental groups and housed in the corresponding clean individual pens with free access to water. Two experimental diets were prepared: RSMTD-1 diet (containing 60% alfalfa silage inoculated with *L. plantarum* MTD/1 and 40% concentrate) and ES24-7 diet (containing 60% alfalfa silage inoculated with *L. plantarum* 24-7 and 40% concentrate). The silage-concentrate mixture was formulated on a DM basis according to NRC [23] to meet the nutritional requirement of dairy goats with a BW of 40 kg (Table 1). The experiment was conducted for 56 d (the first 14 d were for pen and diet acclimatization; the intermediate period (35 d) feeding trial was performed; and the last 7 d were for sampling). Each goat was individually identified and allowed free movement within the pens during the off-feeding time. The amount of silage and concentrate provided to each dairy goat (tied at a fixed bunk position) was recorded twice daily at 07:00 and 17:00 h based on feed refusals (at least 10% refusals on an as-fed basis) to estimate voluntary DM intake (DMI). During the feeding trial, each baled silage was sampled (100 g) after opening and immediately frozen at −20 °C, while the concentrate was collected weekly. All silages and concentrates were manually mixed according to their respective treatments, with 6 replicates per treatment. The goats were manually milked before each feeding time, and the milk yield was recorded. Before milking, the udder was cleaned with warm water and disinfected with 1.0% sodium hypochlorite solution, and teats were monitored for the presence of milk clots to prevent mastitis.

**Table 1:** Ingredients and chemical compositions of experimental diets

Items	Dietary treatment^3^	
	RSMTD-1	ES24-7
Ingredient, % DM		
Alfalfa silage	60	60
Corn grain	22	22
Wheat bran	4	4
Flax oil residue	8	8
Soybean residue	4	4
Sodium bicarbonate	0.4	0.4
Commercial premix^1^	1.6	1.6
Chemical composition, g/kg DM^2^		
CP	175	176
aNDF	331	325
ADF	256	254

^1^Commercial premix provided (per kg of premix): Vitamin A, 625,000 IU; Vitamin D_3_, 200,000 IU; VE, 1,250 IU; Cu, 625 mg; Fe, 9,500 mg; Zn, 3,700 mg; Mn, 3,700 mg; I, 250 mg; Se, 12.5 mg; Co, 50 mg

^2^*DM* Dry matter, *CP* Crude protein, *aNDF* Neutral detergent fiber assayed with a heat-stable amylase and expressed inclusive of residual ash, *ADF* Acid detergent fiber

^3^RSMTD-1, alfalfa silage inoculated with reference strain *L. plantarum* MTD/1; ES24-7, alfalfa silage inoculated with experimental strain *L. plantarum* 24-7

### Milk production and whey antioxidant capacity

Milk samples were mixed daily during the last week (50 to 55 d) at a ratio of 6:4 (v/v, morning:afternoon) for subsequent analysis. The milk pH was measured from the mixed samples using a portable pH meter (PB-10, Sartorius, Goettingen, Germany). Milk samples were divided into three parts. One aliquot was preserved at 4 °C with bronopol tablet (Sigma Aldrich (Shanghai) Trading Co., Ltd., Shanghai, China) until analysis for fat, protein, lactose, casein, total solid (TS), solids-not-fat (SNF), milk urea and milk free fatty acid (FFA) using an Automatic milk composition analyzer (MilkoScan FT1, Foss, Hillerød, Denmark) within 15 d after sampling. The 3.5% fat-corrected milk (3.5% FCM) was calculated according to Hamzaoui et al. [24], and the formula used was: 3.5% FCM = milk yield (kg) × [0.432 + 0.162 × fat (%)]. The energy-corrected milk (ECM, kg/d) was calculated as previously described by Sjaunja et al. [25], using the equation: ECM = milk yield (kg/d) × [38.3 × fat (g/kg) + 24.2 × protein (g/kg) + 16.54 × lactose (g/kg) + 20.7]/3,140. Another sub-sample of milk was freeze-dried for the determination of vitamin A. The extraction method for vitamin A from the freeze-dried milk (1 g) was the same as that for α-tocopherol and β-carotene extraction, as described previously [17]. Vitamin A concentration was detected using an Agilent HPLC 1200 at 326 nm (eluent: 1 mL/min, acetonitrile-dichloromethane-methyl alcohol 7:2:1 (v/v/v); temperature: 25 °C). The third milk aliquot was stored at −20 °C until antioxidant capacity determination. Before measuring antioxidant capacity, the thawed milk sample was pretreated by adding 1 mol/L HCl to reach a pH value of 4.6, then centrifuged at 3,000 × *g* for 20 min. Whey antioxidant activity of milk was determined using commercial assay kits as mentioned earlier.

### Blood analysis and antioxidant determination

Blood samples (4 mL) were collected from the jugular vein of each goat using general vacutainer collection tubes and K_2_-EDTA-containing anticoagulative tubes (5 mL, Jiangxi Glance Medical Equipment Co., Ltd., Nanchang, China) on d 55 of the experimental period. Blood collection tubes with K_2_-EDTA were sent to the local animal hospital immediately for hematological parameters. General vacutainer tubes were subsequently centrifuged at 3,000 × *g* for 15 min to separate serum from the blood. The serum samples were stored in liquid nitrogen and transported to the laboratory for the detection of antioxidant capacity (using the same assay kits as for silage antioxidant activity detection), immunoglobulin levels (IgA, IgG, and IgM), and the concentrations of interleukins (IL-1β, IL-2, IL-4, IL-6, and IL-10), interferon-γ (IFN-γ) and tumor necrosis factor-α (TNF-α). Commercially available enzyme-linked immunosorbent assay (ELISA) kits for goats purchased from Beijing SINO-UK Institute of Biological Technology (Beijing, China) were used for determinations of IgA, IgG, and IgM levels, and IL-1β, IL-2, IL-4, IL-6, IL-10, IFN-γ, and TNF-α concentrations.

### Ruminal fermentation parameters and feces antioxidant capacity

Prior to the morning feeding (56 d), the rumen fluid was sampled from each goat using a clean and flexible esophageal tube. We collected 40 mL of rumen fluid from each goat after discarding the initial 30 mL to prevent saliva contamination. Ruminal pH was immediately measured using a portable pH meter (PB-10, Sartorius, Goettingen, Germany). The collected rumen fluids were filtered through 4-layers of cheesecloth and frozen in liquid nitrogen for subsequent analysis of volatile fatty acid (VFA) and NH_3_-N according to Li et al. [15] and Broderick and Kang [18], respectively. During 52 to 55 d, feces were collected from each goat after daily feeding and subsamples were frozen in liquid nitrogen for further determination of antioxidant activity using assay kits as mentioned earlier.

### Mammary gland sampling and mRNA gene expression analysis

On the last day of the experiment, mammary gland tissue was obtained using a semi-automatic biopsy needle (16 G × 90 mm, TSK Corporation, Tochigi, Japan) according to the methods of Zhang et al. [26] and Tian et al. [10]. Each goat was intravenously injected with a Xylazine Hydrochloride (Jilin Huamu Animal Health Products Co., Ltd., Changchun, China) and subcutaneous injection (local anesthesia at the sampling point) with Procaine (contains penicillin and sodium penicillin, Jilin Huamu Animal Health Products Co., Ltd., Changchun, China) 15 min before sampling. The tissue samples were placed in a cryogenic storage tube (2.0-mL) and immediately frozen in liquid nitrogen. RNA extraction was performed immediately upon reaching the laboratory.

Total RNA was extracted using the TRIzol Plus RNA Purification kit (Thermo Fisher Scientific, Waltham, MA, USA). Subsequently, the RNA purity was detected by measuring the 260/280 absorbance ratio using a NanoDrop Spectrophotometer (ND-2000, Thermo Fisher Scientific), while the integrity of the extracted RNA was observed by verifying the discrete bands of 18S and 28S RNA (1% agarose gel electrophoresis) using an ImageQuant LAS 400 imager (GE Healthcare BioSciences). Subsequently, cDNA was synthesized with a Takara Reverse Transcription kit (RR047A, Takara Biomedical Technology (Beijing) Co., Ltd., Beijing, China).

The relative mRNA abundance of target genes was assayed using quantitative real-time PCR (RT-PCR) amplification by an Applied Biosystems QuantStudio 5 Real-Time PCR System (Thermo Fisher Scientific). The fourteen target genes included NADPH oxidase 4 (*NOX4*), tumor necrosis factor (*TNF*), interferon gamma (*IFNG*), nuclear factor erythroid 2 like 2 (*NFE2L2,* also known as *Nrf2*), beta-carotene oxygenase 1 (*BCO1*), alpha tocopherol transfer protein (*TTPA*), *SOD1*, *SOD2*, *SOD3*, glutathione peroxidase 1 (*GPX1*), *GPX2*, *CAT*, glutathione-disulfide reductase (*GSR*), and heme oxygenase 1 (*HMOX1*). The glyceraldehyde-3-phosphate dehydrogenase (*GAPDH*) gene was used as the housekeeper gene. All gene sequences were obtained from the National Center for Biotechnology Information (NCBI), and the primers of all genes were designed and synthesized by Tsingke Biotechnology Co., Ltd. (Additional file 1: Table S3). The operation and reaction procedures of RT-PCR amplification were set according to the manufacturer’s instruction of the TB Green Premix Ex Taq II (Tli RNaseH Plus) kit (RR820A, Takara Biomedical Technology (Beijing) Co., Ltd., Beijing, China). The cycling conditions were 10 min at 95 °C for pre-denaturation, 40 cycles of 15 s at 95 °C for amplification, and annealing at 60 °C for 30 s. Each gene was run in triplicate for amplification. The relative mRNA expression of target genes was calculated using the 2^−ΔΔCt^ method [27].

### Statistical analysis

The Statistical Package for Social Science (SPSS 21.0, SPSS, Inc., Chicago, IL, USA) software was used for all the statistical analyses. The data were fitted into a general linear model (GLM) for a completely randomized design. The diet treatments were considered as fixed effect, while the animals as random effect. The significant mean differences between the diet treatments were separated using Tukey’s honestly significant difference (HSD) at 5% probability level. Marginal significance was considered between 5% and 10% probability level.

## Results

### Fermentation profile, chemical characteristics, and antioxidant capacity of alfalfa silage

In contrast to the RSMTD-1 silage, the ES24-7 silage exhibited higher acetic and lower propionic acid content, as well as a decreased trend in aNDF content (Table 2). Butyric acid was not detected in the two treated silages. The ES24-7 silage also presented higher α-tocopherol and β-carotene concentrations compared with RSMTD-1 silage. As expected, ES24-7 silage exhibited higher T-AOC, GSH-Px, and CAT activities than RSMTD-1 silage (*P* ≤ 0.001), and CAT activity was not detected in RSMTD-1 silage.

**Table 2:** Fermentation characteristic, chemical composition, and antioxidant capacity of ensiled alfalfa

Items^1^	Treatment^2^		SEM^3^	*P*-value
	RSMTD-1	ES24-7		
Fermentation characteristic				
pH	4.62	4.63	0.008	0.832
Lactic acid, g/kg DM	42.3	43.2	0.33	0.189
Acetic acid, g/kg DM	17.6	18.5	0.13	0.005
Propionic acid, g/kg DM	5.38	4.66	0.091	0.003
Chemical composition				
DM, g/kg FW	401	399	1.6	0.624
WSC, g/kg DM	5.45	5.65	0.149	0.502
CP, g/kg DM	141	147	1.7	0.136
NPN, g/kg TN	363	320	11.6	0.186
NH_3_-N, g/kg TN	43.2	41.2	1.08	0.393
aNDF, g/kg DM	400	393	2.1	0.092
ADF, g/kg DM	279	274	3.5	0.550
Antioxidant capacity				
α-tocopherol, mg/kg DM	19.9	26.3	0.76	0.002
β-carotene, mg/kg DM	159	209	5.3	0.001
T-AOC, mmol/kg FW	7.58	9.26	0.093	< 0.001
SOD, U/g FW	785	659	11.7	< 0.001
GSH-Px, U/g FW	438	488	5.1	0.001
CAT, U/g FW	0.00	9.80	0.218	< 0.001

^1^*DM* Dry matter, *FW* Fresh weight, *WSC* Water-soluble carbohydrates, *CP* Crude protein, *NPN* Non-protein nitrogen, *NH*_*3*_*-N* Ammonia nitrogen, *aNDF* Neutral detergent fiber assayed with a heat-stable amylase and expressed inclusive of residual ash, *ADF* Acid detergent fiber, *T-AOC* Total antioxidant capacity, *SOD* Superoxide dismutase, *GSH-Px* Glutathione peroxidase, *CAT* Catalase

^2^RSMTD-1, alfalfa silage inoculated with reference strain *L. plantarum* MTD/1; ES24-7, alfalfa silage inoculated with experimental strain *L. plantarum* 24-7

^3^*SEM* Standard error of the mean; values for silage represent the means of 6 replicates (*n* = 6)

### Dry matter intake, milk production, and whey antioxidant capacity of dairy goats

The DMI of the dairy goats fed diet containing either RSMTD-1 or ES24-7 silage was not different (*P* > 0.05; Table 3), however, there was a higher significant ECM (*P* = 0.009) and marginally significant (*P* = 0.058) 3.5% FCM in the goats fed with ES24-7 silage. These results could be partly attributed to the higher milk fat (*P* = 0.040) and milk casein (*P* = 0.011) yields of goats fed the ES24-7 silage. Moreover, besides higher percentages of milk fat, casein, TS, and SNF, higher (*P* < 0.05) milk urea, milk FFA, and vitamin A concentrations were also observed in ES24-7 versus RSMTD-1 treatment group. The ES24-7 silage significantly influenced the whey antioxidative enzyme activities of dairy goats (*P* < 0.05; Table 3). The activities of SOD, GSH-Px, and CAT in the whey of goats fed with ES24-7 silage increased remarkably, while GSH-Px was not detected in the whey of goats fed with RSMTD-1 silage. T-AOC in whey did not differ between RSMTD-1 and ES24-7 treatment groups (*P* > 0.05).

**Table 3:** Effect of diets on DMI, milk production, and whey antioxidant capacity in dairy goats

Items^1^	Dietary treatment^2^		SEM^3^	*P*-value
	RSMTD-1	ES24-7		
DMI, kg/d	0.811	0.778	0.043	0.717
pH	6.61	6.50	0.030	0.074
Milk production				
Milk yield, kg/d	1.41	1.39	0.048	0.900
3.5% FCM, kg/d	1.36	1.42	0.014	0.058
ECM, kg/d	1.32	1.40	0.015	0.009
Fat, g/d	46.4	50.3	0.85	0.040
Protein, g/d	52.9	57.7	1.59	0.150
Lactose, g/d	63.0	61.4	0.65	0.241
Casein, g/d	39.7	47.3	1.31	0.011
TS, g/d	162	172	2.4	0.063
SNF, g/d	126	134	2.0	0.062
Milk components				
Fat, %	3.30	3.60	0.061	0.024
Protein, %	3.76	4.14	0.113	0.115
Lactose, %	4.48	4.41	0.046	0.441
Casein, %	2.83	3.39	0.093	0.008
TS, %	11.5	12.3	0.17	0.035
SNF, %	8.97	9.62	0.139	0.035
Milk urea, mg/100 mL	53.3	63.9	1.54	0.004
Milk FFA, mEq/L	0.38	1.61	0.028	< 0.001
Vitamin A, μg/100g	597	712	11.1	< 0.001
3.5% FCM/DMI	1.67	1.83	0.024	0.006
Whey antioxidant				
T-AOC, mmol/L	0.087	0.091	0.006	0.786
SOD, U/mL	14.4	23.9	0.36	< 0.001
GSH-Px, U/mL	0.00	22.6	0.54	< 0.001
CAT, U/mL	0.131	0.602	0.052	0.005

^1^ *DMI* Dry matter intake, *3.5% FCM* 3.5% Fat-corrected milk, *ECM* Energy-corrected milk, *TS* Total solid, *SNF* Solid non-fat, *Milk FFA* Milk free fatty acid, *T-AOC* Total antioxidant capacity, *SOD* Superoxide dismutase, *GSH-Px* Glutathione peroxidase, *CAT* Catalase

^2^ RSMTD-1, dairy goats fed with *L. plantarum* MTD/1 treated alfalfa silage; ES24-7, dairy goats fed with *L. plantarum* 24-7 treated alfalfa silage

^3^*SEM* Standard error of the mean; values for milk represent the means of 10 samples (*n* = 10)

### Blood hematological traits and serum antioxidant capacity of dairy goats

The hematological traits and serum antioxidant capacity are shown in Table 4. Compared with the RSMTD-1 silage diet, feeding ES24-7 silage had minor effects (*P* > 0.05) on the hematological parameters, except for hemoglobin level (Hb, *P* = 0.042) that was higher in the serum of goats fed with ES24-7 versus RSMTD-1 silage. At the same time, marginal significance was observed in blood basophil (BASO) percentage (*P* = 0.068) and MCH (mean corpuscular hemoglobin) content (*P* = 0.094) in the ES24-7 treatment group compared with RSMTD-1 group. The T-AOC, SOD, GSH-Px, and CAT activities in the serum of ES24-7 silage-fed goats were significantly higher (*P* < 0.001) than those in the serum of RSMTD-1 treatment group.

**Table 4:** Blood hematological parameters and serum antioxidant capacity of dairy goats

Items^1^	Dietary treatments^2^		SEM^3^	*P*-value
	RSMTD-1	ES24-7		
Blood hematological parameters				
WBC, 10^9^/L	11.8	12.4	0.45	0.541
NEUT, %	10.1	11.2	0.50	0.275
LYMPH, %	51.8	55.2	1.52	0.287
MONO, %	36.2	32.4	1.32	0.173
EO, %	1.62	0.85	0.392	0.338
BASO, %	0.27	0.34	0.019	0.068
RBC, 10^12^/L	1.61	1.95	0.131	0.201
HCT, %	5.45	6.48	0.379	0.186
Hb, g/L	90.1	101.6	2.65	0.042
MCH, pg	49.8	57.4	2.39	0.094
Serum antioxidant				
T-AOC, mmol/L	0.21	0.30	0.007	< 0.001
SOD, U/mL	62.7	95.2	1.73	< 0.001
GSH-Px, U/mL	102	190	2.5	< 0.001
CAT, U/mL	3.47	7.53	0.215	< 0.001

^1^*WBC* White blood cell, *NEUT* Neutrophil, *LYMPH* Lymphocyte, *MONO* Monocytes, *EO* Eosinophil, *BASO* Basophil, *RBC* Red blood cell, *HCT* Hematocrit, *Hb* Hemoglobin, *MCH* Mean corpuscular hemoglobin, *T-AOC* Total antioxidant capacity, *SOD* Superoxide dismutase, *GSH-Px* Glutathione peroxidase, *CAT* Catalase

^2^RSMTD-1, dairy goats fed with *L. plantarum* MTD/1 treated alfalfa silage; ES24-7, dairy goats fed with *L. plantarum* 24-7 treated alfalfa silage

^3^*SEM* Standard error of the mean; values for blood and serum represent the means of 10 samples (*n* = 10)

### Serum immunoglobulin, proinflammatory and anti-inflammatory cytokines of dairy goats

**Fig. 1:**
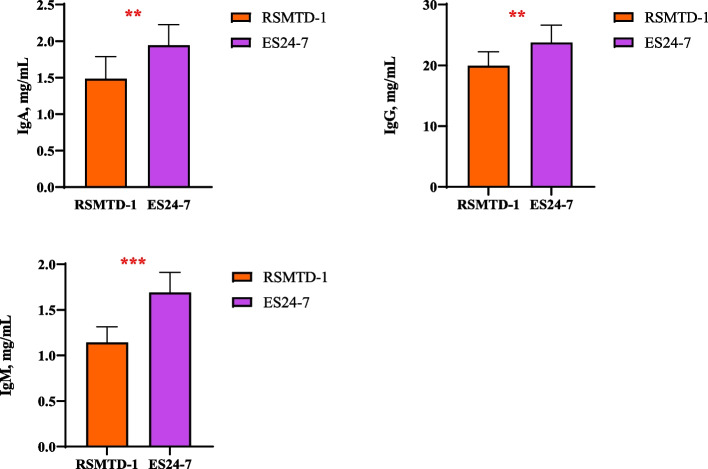
Immunoglobulin concentrations in the serum of dairy goats. Treatment: RSMTD-1, dairy goats fed with *L. plantarum* MTD/1 treated alfalfa silage; ES24-7, dairy goats fed with *L. plantarum* 24-7 treated alfalfa silage. IgA, immunoglobulin A; IgG, immunoglobulin G; IgM, immunoglobulin M. Values for serum represent the means of 10 samples (*n* = 10). Bars indicate standard error of means. ^**^P < 0.01, ^***^P < 0.001

**Fig. 2:**
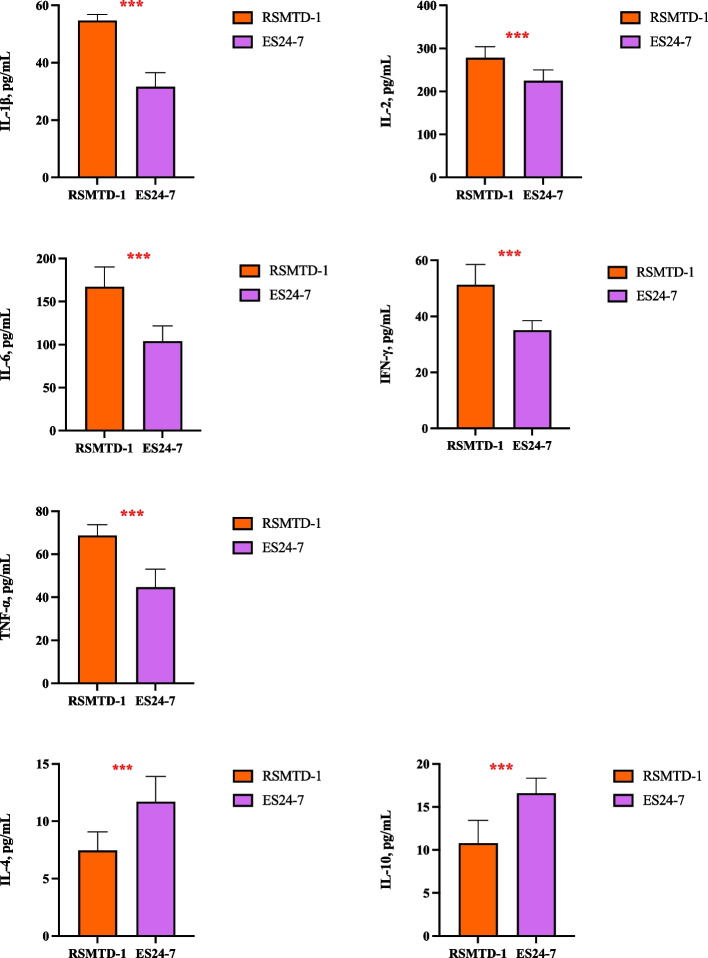
Inflammatory and anti-inflammatory cytokine concentrations in the serum of dairy goats. Treatment: RSMTD-1, dairy goats fed with *L. plantarum* MTD/1 treated alfalfa silage; ES24-7, dairy goats fed with *L. plantarum* 24-7 treated alfalfa silage. IL-1β, interleukin 1β; IL-2, interleukin 2; IL-6, interleukin 6; IFN-γ, interferon-γ; TNF-α, tumor necrosis factor-α; IL-4, interleukin 4; IL-10, interleukin 10. Values for serum represent the means of 10 samples (*n* = 10). Bars indicate standard error of means. ^***^P < 0.001

The goats fed with ES24-7 silage had higher concentrations of IgA, IgG, and IgM (*P* < 0.01) in the serum compared with RSMTD-1 silage-fed goats (Fig. 1). The concentrations of proinflammatory and anti-inflammatory factors in the serum of dairy goats fed with RSMTD-1 and ES24-7 silages are shown in Fig. 2. Lower concentrations (*P* < 0.001) of IL-1β, IL-2, IL-6, IFN-γ, and TNF-α were observed in the serum of dairy goats fed with ES24-7 silage. However, the concentrations of serum IL-4 and IL-10 were higher in the goats fed with ES24-7 versus RSMTD-1 silage (*P* < 0.001).

### Ruminal fermentation parameters and fecal antioxidant capacity of dairy goats

Feeding the two experimental diets showed no effect (*P* > 0.05) on butyrate, valerate, and isovalerate concentrations (Table 5). However, the pH was lower in the rumen fluid of the goats fed with ES24-7 versus RSMTD-1 silage. In addition, higher (*P* < 0.001) total VFA, acetate, and propionate concentrations, and lower (*P* < 0.001) concentrations of isobutyrate and total branched chain volatile fatty acid (BCVFA) were also observed in the rumen of goats fed with ES24-7 silage. Rumen NH_3_-N content was significantly higher (*P* = 0.004) with a marginally significant (*P* = 0.058) ratio of acetate to propionate (AP ratio) in lactating goats fed with ES24-7 silage. Feeding ES24-7 silage had a significant positive impact on the antioxidant capacities in the feces of lactating dairy goats (*P* < 0.001), and increased the fecal T-AOC, SOD, GSH-Px, and CAT activities.

**Table 5:** Ruminal fermentation parameters and feces antioxidant capacity of dairy goats

Items^1^	Dietary treatments^2^		SEM^3^	*P*-value
	RSMTD-1	ES24-7		
Ruminal fermentation				
pH	7.19	6.85	0.016	< 0.001
Total VFA, mmol/L	46.0	62.4	0.80	< 0.001
Acetate, mmol/L	34.6	49.2	0.65	< 0.001
Propionate, mmol/L	5.98	7.74	0.152	< 0.001
Butyrate, mmol/L	3.81	4.13	0.222	0.485
Valerate, mmol/L	0.36	0.32	0.011	0.128
Isobutyrate, mmol/L	0.64	0.46	0.012	< 0.001
Isovalerate, mmol/L	0.55	0.51	0.022	0.356
Total BCVFA, mmol/L	1.20	0.98	0.030	0.005
AP ratio	5.81	6.39	0.135	0.058
NH_3_-N, mg/dL	2.81	4.87	0.272	0.004
Feces antioxidant				
T-AOC, mmol/g protein	0.48	0.55	0.005	< 0.001
SOD, U/mg protein	9.04	11.64	0.204	< 0.001
GSH-Px, U/mg protein	9.63	28.14	1.724	< 0.001
CAT, U/mg protein	0.70	2.17	0.116	< 0.001

^1^*VFA* Volatile fatty acid, *BCVFA* Branched chain volatile fatty acid, *AP ratio* Ratio of acetate to propionate, *NH*_*3*_*-N* Ammonia nitrogen, *T-AOC* Total antioxidant capacity, *SOD* Superoxide dismutase, *GSH-Px* Glutathione peroxidase, *CAT* Catalase

^2^RSMTD-1, dairy goats fed with *L. plantarum* MTD/1 treated alfalfa silage; ES24-7, dairy goats fed with *L. plantarum* 24-7 treated alfalfa silage

^3^*SEM* Standard error of the mean; values for rumen fluid represent the means of 10 samples (*n* = 10)

### Relative mRNA abundance of oxidation- and antioxidant-related genes

**Fig. 3:**
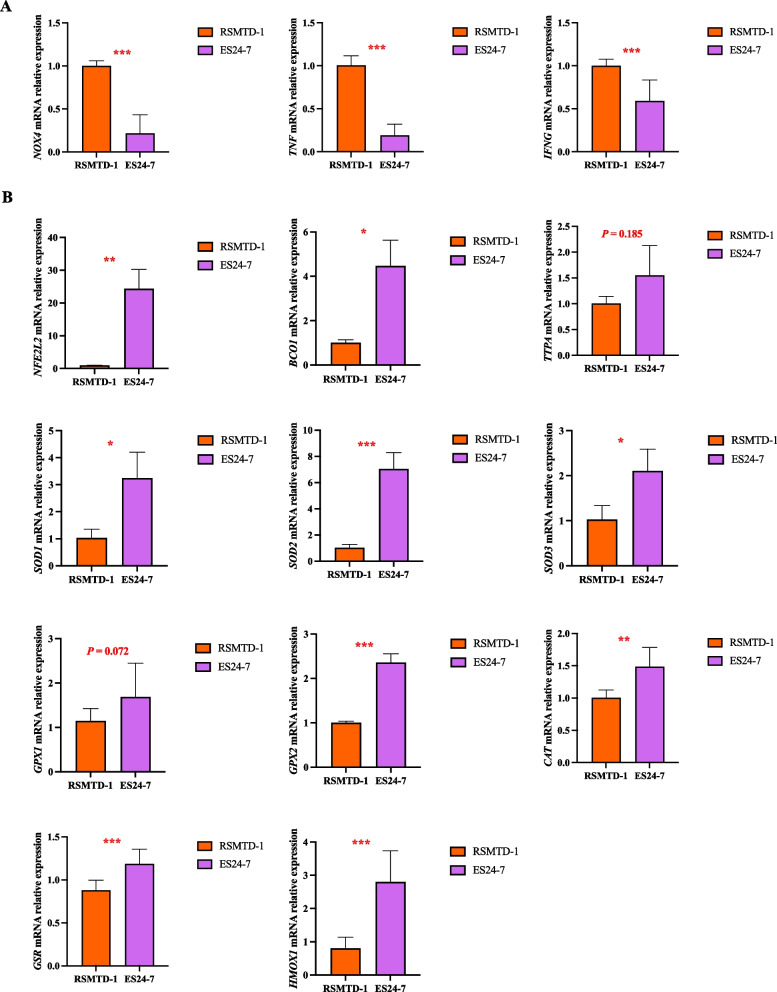
Relative expression of oxidation- (**A**) and antioxidant-related (**B**) genes in the mammary gland sampled from dairy goats. Treatment: RSMTD-1, dairy goats fed with *L. plantarum* MTD/1 treated alfalfa silage; ES24-7, dairy goats fed with *L. plantarum* 24-7 treated alfalfa silage. *NOX4*, NADPH oxidase 4; *TNF*, tumor necrosis factor; *IFNG*, interferon gamma; *NFE2L2*, nuclear factor erythroid 2 like 2; *BCO1*, beta-carotene oxygenase 1; *TTPA*, alpha tocopherol transfer protein; *SOD1*, superoxide dismutase 1; *SOD2*, superoxide dismutase 2; *SOD3*, superoxide dismutase 3; *GPX1*, glutathione peroxidase 1; *GPX2*, glutathione peroxidase 2; *CAT*, catalase; *GSR*, glutathione-disulfide reductase; *HMOX1*, heme oxygenase 1. Values for the mammary gland represent the means of 10 samples (*n* = 10); Bars indicate standard error of means. ^*^P < 0.05, ^**^P < 0.01, ^***^P < 0.001

Goats fed with ES24-7 silage had lower levels (*P* < 0.001) of *NOX4*, *TNF*, and *IFNG* mRNA abundance in the mammary gland than those in the RSMTD-1 silage-fed dairy goats (Fig. 3). RSMTD-1 and ES24-7 silages did not affect (*P* > 0.05) the mRNA expression of *TTPA* and *GPX1* in the mammary gland of dairy goats. The relative mRNA levels of *NFE2L2*, *BCO1*, *SOD1*, *SOD2*, *SOD3*, *GPX2*, *CAT*, *GSR*, and *HMOX1* genes were higher (*P* < 0.05) in the mammary gland of dairy goats fed with the ES24-7 silage than those in the goats fed with RSMTD-1 silage.

## Discussion

The commercial LAB strain *L. plantarum* MTD/1 has been widely used in silage for the improvement of fermentation quality [28–30], while *L. plantarum* 24-7 strain does not only improve the silage fermentation quality but also has a proven ability to improve silage antioxidant status [16, 17]. In the present study, *L. plantarum* 24-7 significantly increased acetic acid and decreased propionic acid content in alfalfa silage, while having no difference in other fermentation characteristics and chemical composition compared with RSMTD-1 silage. The increased acetic acid concentration could improve the silage aerobic stability in the ES24-7 silage [28]. Although propionic acid is also effective in improving aerobic stability [31], its excessive concentration (> 0.5%) in RSMTD-1 silage might have been as an end product of Clostridia, which could have limited the silage fermentation [32]*.* Antioxidant LAB strain or exogenous antioxidant supplements in silage had a remarkable effect on antioxidant bioactive component accumulation [5, 16, 17, 30]. The α-tocopherol and carotenoids concentrations in diets could influence vitamin content in milk, as well as induce positive effects on antioxidant activity and the immune status of animals [33–35]. The higher concentrations of α-tocopherol and β-carotene observed in the *L. plantarum* 24-7 treated silage might be due to the strain’s ability to prevent α-tocopherol and β-carotene from being degraded or to promote biosynthesis of these two substances by microbes in silage [36, 37], which could modulate the OS status. In addition, the changes in T-AOC, GSH-Px, CAT as well as SOD activities of the baled silages inoculated with the antioxidant LAB strain were similar to those observed for silage ensiled in the laboratory silos, which mainly exhibited an increased T-AOC, GSH-Px, and CAT with a decreased SOD [16, 17]. The decrease in SOD value indirectly indicated a lower concentration of ${{\text{O}}}_{2}^{-}$ in ES24-7-inoculated treatment versus the RSMTD-1-inoculated silage. This result might be due to the fact that *L. plantarum* 24-7 has a higher ${{\text{O}}}_{2}^{-}$ scavenging ability relative to *L. plantarum* MTD/1, or it generates less ${{\text{O}}}_{2}^{-}$ in acidic and anaerobic environments at the initial stage of fermentation [38, 39]. The antioxidant enzymes and non-enzyme compounds found in the ES24-7 silage could positively regulate OS and immune performance of dairy goats during lactation.

The feed efficiency (3.5% FCM/DMI) has been remarkably increased by 9.28% (1.83 vs. 1.67) in dairy goats fed diet containing ES24-7 inoculated alfalfa silage. Despite milk yield and DMI being not different, the differences in 3.5% FCM/DMI could be linked to the differences in milk total solids (fat content and protein content) which indicated a positive benefit of feeding ES24-7 inoculated silage than RSMTD-1 inoculated. Silage antioxidant substances fermented by LAB or exogenous antioxidant supplements can improve the antioxidant capacity and immune performance of ruminants, as well as the quality of their products [14, 15, 40]. The cleavage of more β-carotene by *BCO1* and its subsequent conversion into vitamin A was responsible for the higher content of vitamin A in the milk of goats fed *L. plantarum* 24-7 treated silage [41], which is also responsible for the distinctively whiter color of goat milk compared to cows. Moreover, the higher SOD, GSH-Px, and CAT in the whey of goats fed ES24-7-diet could be due to the higher antioxidant enzyme activity, α-tocopherol, and β-carotene contents in ES24-7 silage.

The goats fed with ES24-7-diet exhibited improved oxygen transport capacity due to higher Hb concentration. In addition, ES24-7 silage also increased antioxidant enzyme activities in blood serum which were consistent with the results of Li et al. [15]. In the present study, the higher concentrations of IgA, IgG, and IgM in the serum and improved immune systems of the lactating dairy goats fed with diets containing silage inoculated with *L. plantarum* 24-7 were associated with strong antioxidant activity. The IL-4 and IL-10 have been classified as anti-inflammatory cytokines due to their ability to control inflammation by inhibiting the production and expression of the proinflammatory cytokines (e.g., IL-1, IL-2, IL-6, IFN*,* and TNF) generated by monocytes [42, 43]. In this study, IL-4 and IL-10 concentrations were relatively higher in the ES24-7-fed goats due to the provision of more antioxidant substances by *L. plantarum* 24-7 to antagonize the proinflammatory cytokines, and further alleviate the OS caused by lactation. Hence, the reduction in serum IL-1β, IL-2, IL-6, IFN-γ, and TNF-α of goats fed *L. plantarum* 24-7-inoculated silage which further verified the anti-inflammatory role of the cytokines. Moreover, Li et al. [15] found dairy goats fed with feruloyl esterase-producing LAB inoculated silage to have alleviated OS level through increasing IgA concentration, and reducing the concentrations of TNF-α, IL-2, and IL-6 in serum, which corroborates the findings of the present study.

Higher acetate molar concentration in the rumen of goats fed ES24-7-diet implies higher degradation and fermentation of structural carbohydrates, which indicates the efficiency of the rumen microbial fermentation [15, 44]. Dairy goats fed with ES24-7 diet exhibited a lower but normal-range of rumen pH, facilitating the absorption of VFA by rumen epithelium [45]. Theoretically, a greater rumen propionate yield from the microbial fermentation could contribute to milk lactose synthesis [46], but our study did not observe any significant effect on the milk lactose synthesis. Notably, a lower rumen pH with increased content of NH_3_-N was observed in ES24-7-fed goats, which was inconsistent with the results of earlier studies on the effects of antioxidant compounds on ruminal fermentation [47, 48]. This suggested a rapid release of NH_3_-N from soluble protein degradation or reduced NH_3_-N uptake by rumen microorganisms [49]. Meanwhile, the rumen microbiota is diverse and some of these microorganisms are considered hyper-ammonia-producing bacteria such as asaccharolytic microorganisms [50]. There is a possibility that the observed high NH_3_-N level might be attributed to the rumen microbial modification by *L. plantarum* 24-7, as silage inoculation with LAB has the potential to influence the rumen microbiota. However, rumen microbial analysis is required to substantiate our proposition. Moreover, the higher content of NH_3_-N correlated with high milk urea since NH_3_-N was absorbed and metabolized into urea circulating in the blood and subsequently transported into the mammary gland to end up in milk. To investigate whether the antioxidant substances accumulated in the silage have traversed the rumen and entered the intestinal tract, we determined the fecal antioxidant capacity of the dairy goats in our study. Silage with high antioxidant capacity did not only increased the milk and serum antioxidase activity, but also significantly increased T-AOC, SOD, GSH-Px, and CAT activities in the feces after intestinal metabolism. Despite the higher rumen NH_3_-N in goats fed diet containing ES24-7 inoculated silage, it did not inhibit the antioxidant enzyme activities in the blood serum and feces. The robust antioxidant capacity of *L. plantarum* 24-7 exhibited potential benefits in improving the antioxidant and immune status of lactating dairy goats.

The Nrf2-antioxidant response element (ARE) pathway has been identified to regulate antioxidant activity and suppress redox-sensitive inflammatory pathways [51]. Transcription factor *NFE2L2* is pivotal in inducing the expression of antioxidant proteins and further triggers the expression of downstream target genes *SOD*, *GPX*, *CAT*, *GSR*, and *HMOX1* [7, 51]. In this study, mammary gland *NFE2L2* mRNA expression was up-regulated in dairy goats fed ES24-7-diet, which promoted the expression of downstream transcription factors *SOD1*, *SOD2*, *SOD3*, *GPX2*, *CAT*, *GSR*, and *HMOX1*, as well as inhibited the activities of *NOX4*, *TNF*, and *IFNG*. This was triggered by the higher concentration of the antioxidant enzymes and non-enzyme substances in the ES24-7 silage. Consequently, the induction of the Nrf2/ARE pathway effectively alleviate oxidative damage during lactation, thereby increasing the immune and antioxidant capacity of goats. Thus, feeding diet containing ES24-7 silage had a beneficial effect of inhibiting OS in lactating goats. The increased mRNA level of *BCO1* in the mammary tissue of dairy goats fed with ES24-7 silage, as previously discussed, accounts for the higher vitamin A concentration in the milk. This is attributed to the *BCO1* gene-encoded protein which is identified as the key enzyme in metabolizing vitamin A from β-carotene [41]. *L. plantarum* 24-7-inoculated silage had no effect on the mRNA abundance of *TTPA* in the mammary gland of lactating dairy goats, which might be due to the fact that *TTPA* is majorly expressed in the liver [52, 53]. Feeding diet containing ES24-7 silage also increased the expression of *GPX2* mRNA without affecting the *GPX1* mRNA abundance in the mammary gland, which could be due to the different transcript levels of antioxidant enzyme isoforms of lactating dairy goats [54]. Our results provided evidence that *L. plantarum* 24-7 can regulate the anti-inflammatory and proinflammatory reactions in the organism by modulating the expression of antioxidant- and inflammatory-related genes in the mammary gland, thus protecting cells from oxidative damage and improving the immune function and antioxidant capacity of lactating dairy goats.

## Conclusions

Inoculating alfalfa silage with *L. plantarum* 24-7 exhibited an improved fermentation quality. Additionally, *L. plantarum* 24-7 inoculation also improved antioxidant capacity and the concentrations of α-tocopherol and β-carotene of alfalfa silage. Feeding ES24-7 silage to lactating dairy goats enhanced ruminal acetate and propionate, improved milk quality, as well as immunoglobulins and anti-inflammatory cytokines concentrations in the goats’ serum, which consequently reduced the levels of proinflammatory factors. Feeding alfalfa silage inoculated with *L. plantarum* 24-7 also up-regulated the expression of antioxidant-inducing genes such as *NFE2L2*, *BCO1*, *SOD1*, *SOD2*, *SOD3*, *GPX2*, *CAT*, *GSR*, and *HMOX1* in the mammary gland of dairy goats, and reduced the expression of genes associated with OS (*NOX4*, *TNF*, and *IFNG*), which implies enhanced antioxidant and immunity status of the lactating dairy goats.

### Supplementary Information

**Additional file 1: Table S1.** Nutrient and chemical composition of fresh alfalfa. **Table S2.** The antioxidant activities of *Lactiplantibacillus* *plantarum* MTD/1 and 24-7. **Table S3.** Primer sequences used for quantitative RT-PCR amplifications.

## Data Availability

All data of the research results are within this paper.

## Abbreviations

ADF: Acid detergent fiber
ARE: Antioxidant response element
BCO1: Beta-carotene oxygenase 1
BCVFA: Branched chain volatile fatty acid
BW: Body weight
CAT: Catalase
CP: Crude protein
CFU: Colony-forming units
DIM: Days in milk
DM: Dry matter
DMI: Dry matter intake
ECM: Energy-corrected milk
3.5% FCM: 3.5% Fat-corrected milk
FFA: Free fatty acid
FW: Fresh weight
GAPDH: Glyceraldehyde-3-phosphate dehydrogenase
GLM: General linear model
GPX1: Glutathione peroxidase 1
GSH-Px: Glutathione peroxidase
GSR: Glutathione-disulfide reductase
Hb: Hemoglobin
HMOX1: Heme oxygenase 1
HSD: Tukey’s honestly significant difference
IFN: Interferon
IFNG: Interferon gamma
Ig: Immunoglobulin
IL: Interleukin
*L. plantarum*: *Lactiplantibacillus* *plantarum*
LAB: Lactic acid bacteria
NDF: Neutral detergent fiber
NEUT: Neutrophil
NFE2L2: Nuclear factor erythroid 2 like 2
NH_3_-N: Ammonia nitrogen
NOX4: NADPH oxidase 4
NPN: Non-protein nitrogen
$${{\text{O}}}_{2}^{-}$$: Superoxide anion
OH: Hydroxyl radical
OS: Oxidative stress
ROM: Reactive oxygen metabolites
ROS: Reactive oxygen species
SNF: Solids-not-fat
SOD: Superoxide dismutase
T-AOC: Total antioxidant capacity
TNF: Tumor necrosis factor
TS: Total solid
TTPA: Alpha tocopherol transfer protein
VFA: Volatile fatty acid
WSC: Water-soluble carbohydrate
