# Supplementary material for: Effects of antioxidant-rich Lactiplantibacillus plantarum inoculated alfalfa silage on rumen fermentation, antioxidant and immunity status, and mammary gland gene expression in dairy goats
Source: J Anim Sci Biotechnol. 2024 Jan 22;15:9. doi: 10.1186/s40104-023-00977-3 (PMC10802014; doi:10.1186/s40104-023-00977-3)
Supplement: Supplementary file 1 — Additional file 1: Table S1. Nutrient and chemical composition of fresh alfalfa. Table S2. The antioxidant activities of Lactiplantibacillus plantarum MTD/1 and 24-7. Table S3. Primer sequences used for quantitative RT-PCR amplifications. [file 40104_2023_977_MOESM1_ESM.docx]

**Supplementary information**

**Additional file 1:** Table S1: Nutrient and chemical composition of fresh alfalfa. Table S2: The antioxidant activities of *Lactiplantibacillus plantarum* MTD/1 and 24-7. Table S3: Primer sequences used for quantitative RT-PCR amplifications.

**Table S1** Nutrient and chemical composition of fresh alfalfa

| Items^1^ | Mean ± SD^2^ |
| --- | --- |
| DM, g/kg FW | 403 ± 6.04 |
| pH | 6.62 ± 0.05 |
| WSC, g/kg DM | 20.4 ± 0.64 |
| CP, g/kg DM | 155 ± 2.56 |
| NPN, g/kg TN | 173 ± 0.90 |
| NH_3_-N, g/kg TN | 16.1 ± 0.76 |
| aNDF, g/kg DM | 405 ± 5.17 |
| ADF, g/kg DM | 286 ± 12.6 |
| *α*-tocopherol, mg/kg DM | 19.7 ± 1.34 |
| *β*-carotene, mg/kg DM | 146 ± 5.95 |

^1^DM, dry matter; FW, fresh weight; WSC, water-soluble carbohydrates; CP, crude protein; NPN, non-protein nitrogen; NH_3_-N, ammonia nitrogen; aNDF, neutral detergent fiber assayed with a heat-stable amylase and expressed inclusive of residual ash; ADF, acid detergent fiber.

^2^Data are presented with means ± standard deviation (SD) from three independent experiments.

**Table S2** The antioxidant activities of *Lactiplantibacillus plantarum* MTD/1 and 24-7

| Item^1^ | MTD/1 | 24-7 |
| --- | --- | --- |
| 2 mmol/L H_2_O_2_ | 0.148 | 1.528 |
| FS |  |  |
| DPPH, % | 69.1 ± 1.01 | 87.1 ± 0.03^2^ |
| ·OH, % | 41.3 ± 0.04 | 62.9 ± 0.09 |
| $O_{2}^{-}$, % | 11.6 ± 0.07 | 25.4 ± 0.07 |
| T-AOC, U/mL | 24.9 ± 0.08 | 31.1 ± 0.03 |
| SOD, U/mL | 47.1 ± 1.00 | 65.5 ± 0.01 |
| GSH-Px, U/mL | nd | nd |
| CAT, U/mL | nd | nd |
| IC (1×10^9^ CFU/mL) |  |  |
| T-AOC, U/mL | 0.19 ± 0.00 | 0.56 ± 0.00 |
| SOD, U/mL | 7.76 ± 0.07 | 21.2 ± 0.01 |
| GSH-Px, U/mL | 2.26 ± 0.01 | 18.5 ± 0.05 |
| CAT, U/mL | 0.16 ± 0.00 | 0.65 ± 0.04 |
| CFE (1×10^9^ CFU/mL) |  |  |
| T-AOC, U/mL | 0.99 ± 0.01 | 2.90 ± 0.02 |
| SOD, U/mL | 2.97 ± 0.03 | 13.1 ± 0.15 |
| GSH-Px, U/mL | 5.86 ± 0.10 | 22.1 ± 0.01 |
| CAT, U/mL | 0.09 ± 0.00 | 0.41 ± 0.01 |

^1^2 mmol/L H_2_O_2_, optical density value of strains in concentration of 2 mmol/L H_2_O_2_; FS, fermentation supernatant; DPPH, 2, 2-diphenyl-1-picrylhydrazyl free radical scavenging capacity; ·OH, hydroxyl radical scavenging ability; $O_{2}^{-}$, superoxide anion scavenging ability; T-AOC, total anti-oxidation competence; SOD, superoxide dismutase; GSH-Px, Glutathione peroxidase; CAT, catalase; IC, intact cell; CFE, cell-free extract; nd, not detected.

^2^Data are presented with means ± standard deviation (SD) from three independent experiments.

**Table S3** Primer sequences used for quantitative RT-PCR amplifications

| Gene^1^ | Primer sequences (5’-3’)^2^ | Accession number | Product size, bp |
| --- | --- | --- | --- |
| *NFE2L2* | (F) CTGTTCTCTGCTGTCAAGGGA | NM_001314327.1 | 221 |
|  | (R) ACTCGCCGGTCTCTTCATCT |  |  |
| *BCO1* | (F) CTGAACACGGACGAGCATCT | XM_018061761.1 | 202 |
|  | (R) TTGTATCTGGTCTCGCCCAC |  |  |
| *TTPA* | (F) CTGAAGGCGGGCTATGTCG | XM_018058466.1 | 142 |
|  | (R) TAAGCTCCGACGTGATGAGAC |  |  |
| *SOD1* | (F) AGAGAGGCATGTTGGAGACC | NM_001285550.1 | 160 |
|  | (R) TCCACCTCTGCCCAAGTCAT |  |  |
| *SOD2* | (F) ACCCAAAGGGGAATTGCTGG | XM_018053428.1 | 227 |
|  | (R) CATGCTCCCACACGTCAATC |  |  |
| *SOD3* | (F) CTTCTTCCACCTTGAGGGCTT | NM_001285675.1 | 123 |
|  | (R) GACATCGGGTTGTAGTGCGG |  |  |
| *GPX1* | (F) TCCTTGTTCTTCGAGTCCGC | XM_005695962.3 | 209 |
|  | (R) CCTCAGAGCGATGCTACGTT |  |  |
| *GPX2* | (F) CTCAAGTATGTCCGCCCTGG | XM_005685982.3 | 135 |
|  | (R) CGGGTCGTCATAAGGGTAGG |  |  |
| *GSR* | (F) ACACTGCTGTCCACTCTGAA | XM_018041988.1  XM_018041989.1 | 111 |
|  | (R) TCACATAGGCATCCCGCTTT |  |  |
| *CAT* | (F) CTGGAACATAGGACCCGCTT | XM_005690077.3 | 137 |
|  | (R) GCAATGTTCTCACACAGGCG |  |  |
| *HMOX1* | (F) CACCAAGCGCTATGTTCAGC | NM_001285567.1 | 201 |
|  | (R) TTGGTGGCACTGGCGATATT |  |  |
| *NOX4* | (F) GGGATTGTGTCTAAGCAGAGC | XM_005699426.3  XM_018043547.1 | 136 |
|  | (R) CAATCTTCTGGTTCTCCGGCT |  |  |
| *TNF* | (F) CCCAGAGGGAAGAGCAGTC | NM_001286442.1 | 168 |
|  | (R) TGAGGGCATTGGCATACGAG |  |  |
| *IFNG* | (F) GATCCAGCGCAAAGCCATAAA | NM_001285682.1 | 109 |
|  | (R) TCTCCGGCCTCGAAAGAGATT |  |  |
| *GAPDH* | (F) GTCCGTTGTGGATCTGACCTG | XM_005680968.3 | 165 |
|  | (R) AAGGTAGAAGAGTGAGTGTCGC |  |  |

^1^*NFE2L2*: nuclear factor erythroid 2 like 2; *BCO1*, beta-carotene oxygenase 1; *TTPA*, alpha tocopherol transfer protein; *SOD1*, superoxide dismutase 1; *SOD2*, superoxide dismutase 2; *SOD3*, superoxide dismutase 3; *GPX1*, glutathione peroxidase 1; *GPX2*, glutathione peroxidase 2; *GSR*, glutathione-disulfide reductase; *CAT*, catalase; *HMOX1*, heme oxygenase 1; *NOX4*, NADPH oxidase 4; *TNF*, tumor necrosis factor; *IFNG*, interferon gamma; *GAPDH*, glyceraldehyde-3-phosphate dehydrogenase.

^2^F: Forward, R: Reverse.
